# Optimization Temperature Programming of Microwave-Assisted Synthesis ZnO Nanoneedle Arrays for Optical and Surface-Enhanced Raman Scattering Applications

**DOI:** 10.3390/nano12223989

**Published:** 2022-11-12

**Authors:** Tung-Hao Chang, Yu-Cheng Chang, Chung-I Lee, Ying-Ru Lin, Fu-Hsiang Ko

**Affiliations:** 1 Department of Radiation Oncology, Changhua Christian Hospital, Changhua 50006, Taiwan; 2 Department of Radiological Technology, Yuanpei University, Hsinchu 30015, Taiwan; 3 Department of Medical Imaging and Radiological Sciences, Central Taiwan University of Science and Technology, Taichung 40601, Taiwan; 4 Department of Materials Science and Engineering, Feng Chia University, Taichung 40724, Taiwan; 5 Department of Materials Science and Engineering, National Yang Ming Chiao Tung University, Hsinchu 30010, Taiwan

**Keywords:** microwave-assisted synthesis, ZnO nanoneedle arrays, temperature programming, antireflection, surface-enhanced Raman scattering

## Abstract

This study used a rapid and simple microwave-assisted synthesis method to grow ZnO nanoneedle arrays on the silicon substrate with the ZnO seed layer. The effects of reaction temperature and time on the lengths of ZnO nanoneedle arrays were investigated. The appropriate temperature programming step can grow the longer ZnO nanoneedle arrays at the same reaction time (25 min), which is 2.08 times higher than without the temperature programming step. The geometry of the ZnO nanoneedle arrays features a gradual decrease from the Si substrate to the surface, which provides an excellent progressive refractive index between Si and air, resulting in excellent antireflection properties over an extensive wavelength range. In addition, the ZnO nanoneedle arrays exhibit a suitable structure for uniform deposition of Ag nanoparticles, which can provide three-dimensional hot spots and surface active sites, resulting in higher surface-enhanced Raman scattering (SERS) enhancement, high uniformity, high reusability, and low detection limit for R6G molecule. The ZnO/Ag nanoneedle arrays can also reveal a superior SERS-active substrate detecting amoxicillin (10^−8^ M). These results are promising for applying the SERS technique for rapid low-concentration determination in different fields.

## 1. Introduction

A fast and easy synthesis method is important to allow more accurate experiments in a short reaction time without releasing heat in the environment [1]. Furthermore, microwave-assisted synthesis is considered a more efficient way to control heating in many processes because it requires less energy than traditional synthesis methods [2,3]. The main reason is ascribed to only providing uniform heating to the reaction material throughout the reaction vessel, resulting in fast and uniform heat transfer [4,5]. Therefore, there is no problem with wasting energy in the process [6]. In addition, the related research and application of microwave-assisted synthesis is an emerging green chemistry method [7,8]. It creates one of the best ways to rapidly fabricate materials with nanoscale dimensions and enables control of matter at the nanoscale [2,9]. The main advantages of microwave-assisted synthesis for preparing nanostructures are high reaction rate, high yield, high purity, controllable reaction conditions, and high reproducibility [10,11]. So far, microwave-assisted synthesis has been widely used to prepare organic or inorganic nanostructures, such as polymer nanocomposites [12,13], metal nanostructures [14,15], semiconductor nanostructures [16,17], carbon-based nanostructures [4,18,19], nanoporous structures [20], etc.

Researchers have received the most attention from zinc oxide (ZnO) nanostructures rather than other semiconductor nanostructures [21,22]. ZnO is one of the most widely used functional semiconductor materials. ZnO is a wide direct band gap (3.37 eV), a large electron binding energy (60 meV), and an appropriate optical refractive index (*n* = 1.99), which makes it widely used in optical and electronic applications [23,24,25]. Recently, ZnO nanostructures have been used in light-emitting diodes (LEDs), nanolasers, thin film transistors (TFTs), solar cells, photonic crystals, photocatalysts, and surface-enhanced Raman scatting [26,27,28]. Therefore, ZnO nanostructures are one of the most frequently studied and applied semiconductor materials [29,30]. Many methods have been used to synthesize ZnO nanostructures, including chemical vapor deposition, thermal evaporation, molecular beam epitaxy, hydrothermal, pulsed laser deposition, wet chemical, and microwave-assisted synthesis [31,32]. The microwave-assisted synthesis method for the growth of ZnO nanostructures mainly uses water as the dipole solvent [33]. Under the influence of the rapidly changing alternating electric field, rotation, friction, and collision of water molecules generate heat [33,34]. Therefore, it can be used to prepare ZnO nanostructures rapidly [35].

The antireflection coating plays an important role in increasing the conversion efficiency of solar cells by enhancing the collection of incident light at the interface between the air and the top of the solar cells [35]. Different ZnO nanostructure arrays have recently been very suitable as high-performance antireflection coatings [36,37,38]. The main reasons are high transparency, proper refractive index, and applicable fabrication process [39]. For example, using an antireflection coating with needle-like ZnO nanowire arrays as a silicon substrate, a low reflectivity comparable to that of a SiNx layer can be achieved [40]. In our previous work, ZnO nanoneedle arrays can provide excellent impedance matching between Si and air by gradually decreasing the effective refractive index away from the surface, resulting in excellent antireflection performance over a larger wavelength range [41]. However, no reports used temperature programming to control the fabrication of ZnO nanoneedle arrays as highly efficient antireflection and SERS substrates by microwave-assisted synthesis.

In this study, the controllable temperature programming step can be used to grow ZnO nanoneedle arrays on the silicon substrate with the ZnO seed layer at a short reaction time of 25 min by the microwave-assisted synthesis method. As a result, ZnO nanoneedle arrays with excellent geometry can be used in the high-performance antireflection coating layer. In addition, ZnO nanoneedle arrays can construct a three-dimensional SERS substrate by depositing Ag nanoparticles in the potential applications for environmentally friendly and economical chemical or drug detection.

## 2. Materials and Methods

### 2.1. Fabrication of ZnO Seed Layer

**Figure 1:**
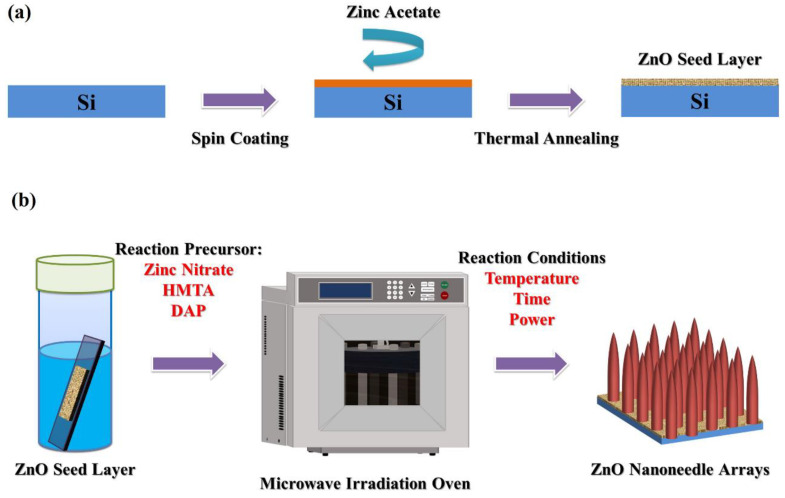
The schematically illustrates the fabrication of (**a**) ZnO seed layer and (**b**) ZnO nanoneedle arrays.

The silicon substrates (3 cm × 3 cm) were immersed in ethanol and cleaned via an ultrasonic vibrator for 10 min to remove particles and organic contaminants from the surface of the substrates. Next, the ZnO seed film was fabricated via spin coating a layer of solution of 20 mM zinc acetate dihydrate (97%, Alfa Aesar) in ethanol and then thermal annealing the substrates at 80 °C for 3 min and 350 °C for 20 min. Finally, the silicon substrates with ZnO seed layer were cut into 1 cm × 3 cm for the growth of ZnO nanoneedle arrays. The fabrication process of the ZnO seed layer is illustrated in Figure 1a.

### 2.2. Fabrication of ZnO Nanoneedle Arrays

The ZnO nanoneedle arrays were fabricated on the silicon substrates with a ZnO seed layer by microwave irradiation in 40 mL of an aqueous solution containing 10 mM zinc nitrate hexahydrate (98%, Alfa Aesar), 10 mM hexamethylenetetramine (HMTA, 99%, Alfa Aesar) and 0.4 mL 1,3-diaminopropane (DAP, 98%, Alfa Aesar). The silicon substrate with a ZnO seed layer (1 cm × 3 cm) was pasted on a Teflon sheet, placed in a sealed Teflon vessel (100 mL) containing the above reaction solution, and heated in a microwave irradiation oven under different reaction temperatures and times. The microwave irradiation oven was performed by a CEM^®^ microwave reactor (MARS 5 Press, USA). The deposition reaction was carried out in a sealed Teflon vessel and operated at a frequency of 2.45 GHz. The temperature control system for MARS 5 Press is the RTP-300 Plus (optic temperature probe) which monitors and controls temperature conditions inside sample vessels from −40–250 °C. A feedback signal from the RTP-300 Plus probe to the system magnetron adjusts the microwave power output to maintain the selected temperature parameter. The fabrication process of the ZnO nanoneedle arrays is illustrated in Figure 1b.

### 2.3. Characteristics

The surface morphologies of ZnO nanoneedle arrays were examined with a field-emission scanning electron microscopy (FESEM, JEOL JSM-6700F, Japan) operating at a 15 kV accelerating voltage. A transmission electron microscopy (TEM, JEOL-2010, Japan) operating at 200 kV was used to examine the microstructures. The crystalline structure of the ZnO nanoneedle arrays was determined using the X-ray powder diffraction method (Shimadzu XRD-6000, CuKα1 radiation (λ = 0.1505 nm), Japan). The photoluminescence (PL, Taiwan) spectroscopy was performed using a 325 nm He−Cd laser as the excitation source. The reflection spectra were obtained with an ultraviolet/visible/near-infrared spectrophotometer (UV/VS/NIR, Hitachi Model U-4100, Japan).

### 2.4. SERS Testing

ZnO nanoneedle arrays deposited Ag nanoparticles to form ZnO/Ag nanoneedle arrays by an ion-beam sputtering system at a ∼3.7 × 10^−6^ Torr pressure. Using the rhodamine 6G (R6G) molecule as the chemical target, the SERS response of ZnO/Ag nanoneedle arrays under the optimized temperature programming step was evaluated. R6G molecule was dissolved in deionized water. The ZnO/Ag nanoneedle arrays were immersed in target solutions of different concentrations for 1 h at room temperature in the dark and then dried by air blowing. A Raman microscope system (MRI532S, Protrustech, Taiwan) equipped with a 532 nm laser was used to acquire SERS spectra. For the Raman measurement conditions, the laser irradiation power and the detector integration time were 1 mW and 0.15 s, respectively.

## 3. Results and Discussion

**Figure 2:**
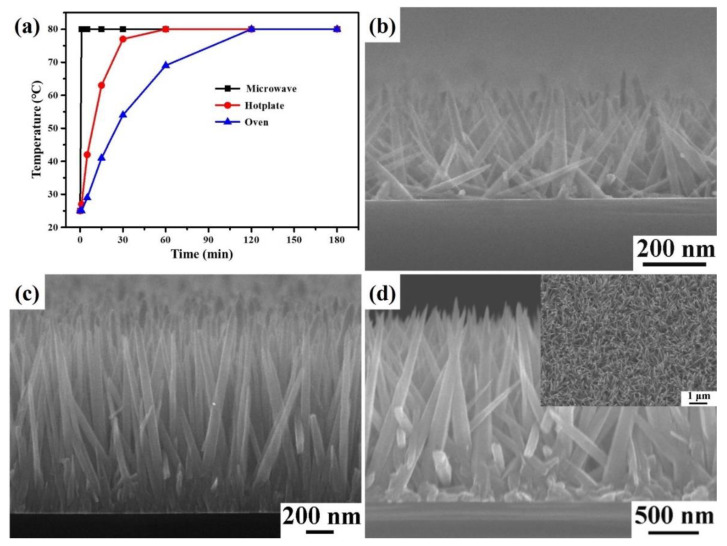
(**a**) The heating curves of microwave irradiation oven, hotplate, and oven. (**b**–**d**) The cross-sectional FESEM images of the ZnO nanoneedle arrays were fabricated on the silicon substrates with the ZnO seed layer at different reaction times. The reaction times are (**b**) 1, (**c**) 15, and (**d**) 60 min, respectively.

In past studies, microwave-assisted synthesis has been highly efficient in shortening the reaction time and increasing the yield [42]. Figure 2a shows the temperature-corresponding time curve of the microwave irradiation oven, hotplate, and oven used to heat 40 mL of the ZnO reaction precursor (10 mM zinc nitrate hexahydrate, 10 mM HMTA, and 0.4 mL DAP) for 180 min. The microwave irradiation oven is observed to heat the ZnO reaction precursor to 80 °C at 1 min. It takes 60 min and 90 min to heat the ZnO reaction precursor to 80 °C using a hot plate and an oven, respectively. Hotplate and oven require thermal diffusion and convection to heat the reactants, but microwave heating only provides uniform heating of the reactants in the entire reaction vessel, thus enabling rapid and direct heating. Furthermore, microwave-assisted synthesis has the advantage of rapidly, accurately, and directly heating the reaction solution, avoiding unnecessary energy waste for energy saving and carbon reduction. Figure 2b–d shows the cross-sectional FESEM images of ZnO nanoneedle arrays fabricated on the silicon substrate with a ZnO seed layer under different reaction times (heating time 1 min + holding time). The reaction times are 1, 15, and 60 min, respectively. The average lengths of ZnO nanoneedle arrays are 0.379 ± 0.333, 1.355 ± 0.289, and 2.995 ± 0.476 μm, respectively. With the gradual increase of the reaction time, the lengths of ZnO nanoneedle arrays also tend to increase gradually. From the inset FESEM image in Figure 2d, it can be found that the size of the ZnO nanoneedle arrays is relatively uniform.

**Figure 3:**
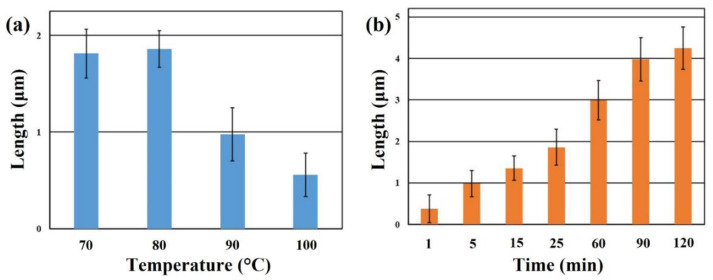
The average lengths of ZnO nanoneedle arrays at different (**a**) reaction temperatures and (**b**) reaction times (reaction time: 1 min (heating time) + holding time).

To more effectively explore the effect of microwave reaction conditions on the growth of ZnO nanoneedle arrays. Herein, the microwave reaction temperature and time were investigated. Figure 3a reveals the lengths of ZnO nanoneedle arrays grown at different reaction temperatures (microwave settings: heating time = 1 min and holding time = 24 min). With the increase in reaction temperature, the lengths of ZnO nanoneedle arrays increased first and then decreased. This phenomenon shall be ascribed to the accelerated consumption of ZnO reaction precursors at the high reaction temperature, which makes it impossible to continue to grow ZnO nanoneedle arrays on the substrate effectively. In addition, the longest ZnO nanoneedle arrays can be grown at the reaction temperature of 80 °C. Therefore, the appropriate reaction temperature can be beneficial to grow longer ZnO nanoneedle arrays. Figure 3b reveals the lengths of ZnO nanoneedle arrays grown at different reaction times (microwave settings: heating time = 1 min and different holding time) at the reaction temperature of 80 °C. With the prolongation of the reaction time, the lengths of the grown ZnO nanoneedle array also tend to increase gradually. This result demonstrates that prolonging the reaction time can increase the lengths of the ZnO nanoneedle arrays.

**Figure 4:**
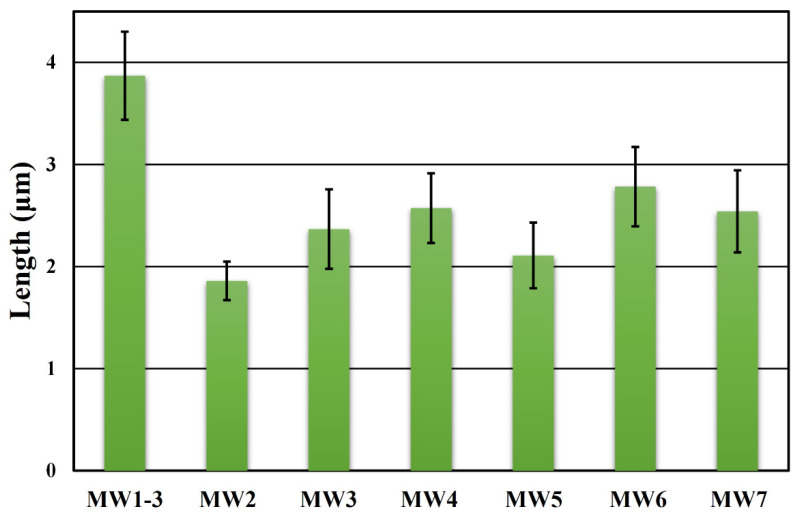
The average lengths of ZnO nanoneedle arrays at different temperature programming steps.

However, increasing the lengths of ZnO nanoneedle arrays only by increasing the reaction time cannot highlight the advantages of microwave-assisted synthesis for fast and accurate heating. Therefore, this study further explored the effect of growing ZnO nanoneedle arrays under different temperature programming steps. The reaction conditions for all microwave temperature programming steps are organized as shown in Table 1. All heating and reaction times (heating and holding times) are controlled at 1 min and 25 min, respectively. Figure 4 reveals the lengths of ZnO nanoneedle arrays grown at different temperature programming steps. The longest ZnO nanoneedle arrays can be grown at the temperature programming steps (microwave settings (MW1-3): heating time = 1 min and holding time = 4 min for 80 °C, heating time = 1 min and holding time = 9 min for 90 °C, and heating time = 1 min and holding time = 9 min for 100 °C). Therefore, changing the temperature and holding time can be beneficial to grow longer ZnO nanoneedle arrays at the same reaction time. When comparing the microwave parameter settings of MW1-3 and MW3, it can be found that the reaction temperature raised too high, the reaction precursor rapidly depleted, and the lengths of ZnO nanoneedle arrays were reduced. The same phenomenon can also be observed in MW6 and MW7. The higher reaction temperature causes the problem of rapid reaction precursor consumption to reduce the lengths of ZnO nanoneedle arrays at the same holding time of 5 min. Comparing MW4 and MW5, it can be found that the holding time of 14 min at 90 °C is more conducive to the growth of ZnO nanoneedle arrays than at 100 °C. Therefore, appropriate reaction temperature and holding time can be beneficial to grow the longest ZnO nanoneedle arrays.

**Figure 5:**
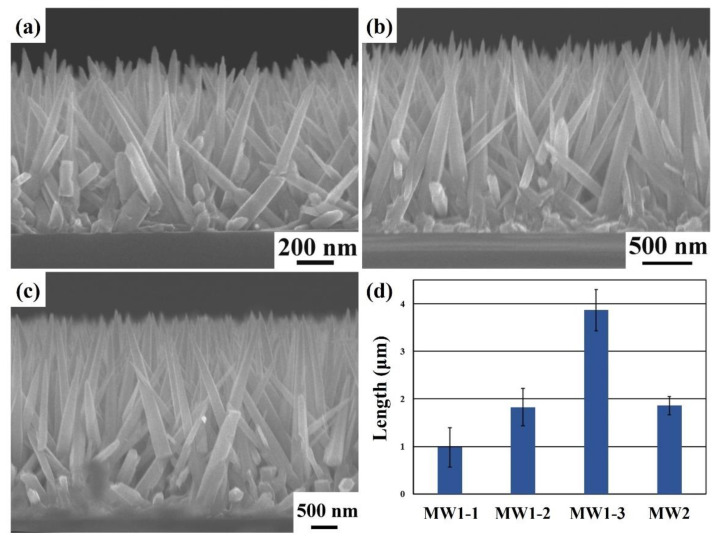
(**a**–**c**) The cross-sectional FESEM images of the ZnO nanoneedle arrays were fabricated on the silicon substrates with the ZnO seed layer at the different optimized temperature programming steps. (**d**) The average lengths of ZnO nanoneedle arrays at different temperature programming steps (MW1-1, MW1-2, MW1-3, and MW2).

Figure 5a–c reveals that the cross-sectional FESEM images of ZnO nanoneedle arrays were grown at the different optimized temperature programming steps. The different optimized temperature programming steps are MW1-1, MW1-2, and MW1-3. The average lengths of ZnO nanoneedle arrays are 0.981 ± 0.415, 1.828 ± 0.396, and 3.869 ± 0.432 μm, respectively. The average diameters of ZnO nanoneedle arrays are 75.2 ± 25.5, 145.6 ± 32.3, and 157.2 ± 61.7 nm, respectively. The lengths and diameters of ZnO nanoneedle arrays tend to increase with the temperature programming steps. Figure 5d reveals the lengths of ZnO nanoneedle arrays grown at different optimized temperature programming steps and MW2 (microwave settings: heating time = 1 min and holding time = 24 min for 80 °C). The average lengths of ZnO nanoneedle arrays (MW2) are 1.859 ± 0.189 μm. The average lengths of ZnO nanoneedle arrays (MW1-3) are about 2.08 times higher than MW2 by controlling different temperature programming steps. This result can also prove that the temperature programming process facilitates the growth of longer ZnO nanoneedle arrays in a short time. In addition, the repeatability of the optimized temperature programming step on the lengths of ZnO nanoneedle arrays at different times has been shown in Figure S2. It can be seen that the lengths of different repeatability are not a very significant change. This result can also be verified that this reaction condition is conducive to the growth of longer ZnO nanoneedle arrays.

**Figure 6:**
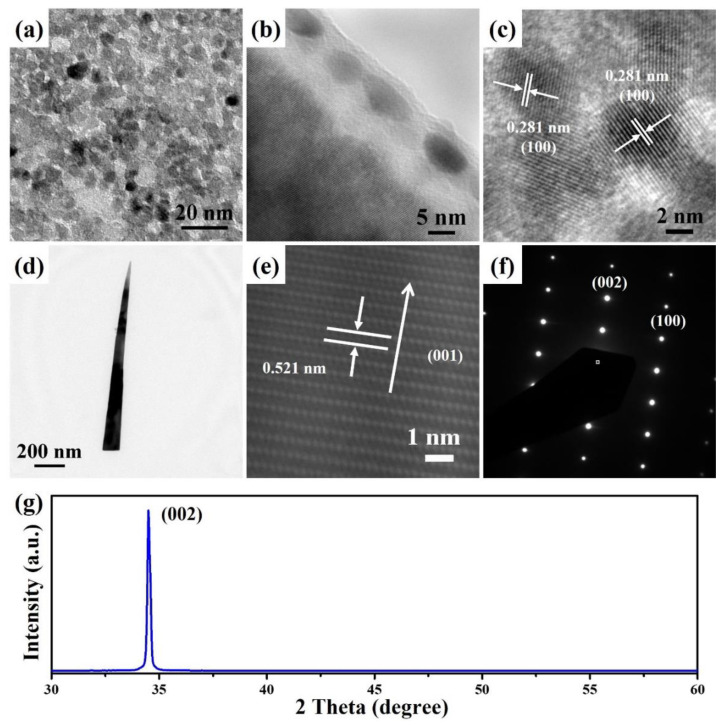
The (**a**) top-view TEM, (**b**) cross-sectional TEM, and (**c**) HRTEM images of a ZnO seed layer were fabricated on the silicon substrate. The (**d**) TEM, (**e**) HRTEM, and (**f**) SAED pattern images of a ZnO nanoneedle (MW1-3). (**g**) XRD spectrum of ZnO nanoneedle arrays was fabricated on the silicon substrate with the ZnO seed layer (MW1-3).

The ZnO seed layer was examined with TEM to understand its microstructure and formation mechanism. A ZnO seed layer can be directly fabricated on the silicon substrate by a facile spin coating and thermal annealing process. Figure 6a shows the top-view TEM image of ZnO seeds with sizes of 4–9 nm. The thickness of a ZnO seed layer is about 5 nm, as shown in Figure 6b. A lattice spacing measurement of 0.281 nm can be detected from the high-resolution TEM (HRTEM) image in Figure 6c, which corresponds to the (100) diffraction plane of the hexagonal phase ZnO (JCPDS no. 89–0511). Figure 6d shows the TEM image of a ZnO nanoneedle grown at the temperature programming step of MW1-3. The morphology of the ZnO nanostructure is the same as those observed by SEM, showing a needle-like structure. Figure 6e,f reveal the HRTEM image and the corresponding selected area electron diffraction (SAED) pattern taken from an individual ZnO nanoneedle, which exhibits the lattice spacing of 0.521 nm and the (010) zone-axis SAED pattern. These results indicate that ZnO nanoneedle is grown along the *c*-axis of the (001) direction. In addition, the overall crystallinity of the ZnO nanoneedle arrays was analyzed by X-ray powder diffraction. The strong and sharp diffraction peak corresponding to the (002) crystal plane of hexagonal phase ZnO indicates that the preferred growth direction of ZnO nanoneedle arrays is in the (001).

**Figure 7:**
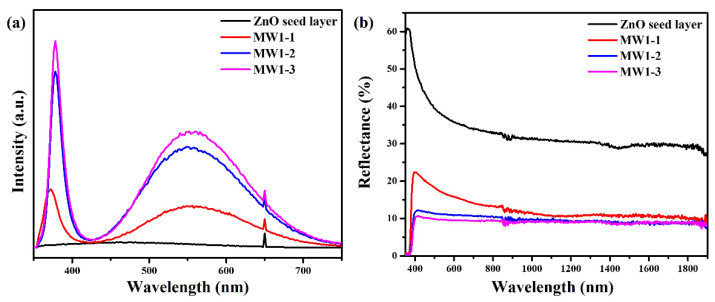
(**a**) PL and (**b**) reflectance spectra of ZnO nanoneedle arrays were fabricated on the silicon substrates with the ZnO seed layer at the optimized temperature programming step.

Photoluminescence (PL) is a helpful technique to characterize the optical emission properties of nanostructures. All PL spectra were measured at room temperature. The influence of the optimized temperature programming step on PL spectra of MW1-1, MW1-2, and MW1-3 is investigated and revealed in Figure 7a. For the ZnO seed layer, no emission peak was measured. However, the PL spectrum of ZnO nanoneedle arrays (MW1-1) revealed a sharp UV emission at 372 nm (3.33 eV) and a broad visible emission at 556 nm (2.23 eV), which were near-bandgap emissions and intrinsic defect structures (such as oxygen vacancies), respectively [43]. For ZnO nanoneedle arrays (MW1-2), two strong peaks are at 378 nm (3.28 eV) and 558 nm (2.22 eV). For ZnO nanoneedle arrays (MW1-3), two very strong peaks are at 378 nm (3.28 eV) and 560 nm (2.21 eV). Compared with MW1-1, the UV emission of MW1-2 and MW1-3 exhibited a significant red-shifted emission. This red-shifted emission can be ascribed to the reverse quantum confinement effect caused by the increase in the sizes of ZnO nanoneedle arrays [44,45].

Figure 7b reveals thereflectance spectra of the ZnO nanoneedle arrays fabricated by the optimized temperature programming step. Compared to the ZnO seed layer and ZnO nanoneedle arrays grown with different optimized temperature programming steps, the importance of ZnO morphology for antireflection properties can be found. The lengths and antireflection performance also tend to increase with the increase of the optimized temperature programming steps. The morphologies of the ZnO nanoneedle arrays exhibit tapered and longer lengths, leading to the best antireflection performance with the gradual decrease in the refractive index of the Si substrate/ZnO/ambient air. In previous studies, ZnO nanostructures with broadband and omnidirectional antireflection coatings have greatly enhanced the performance of optical and electronic devices [37,41].

**Figure 8:**
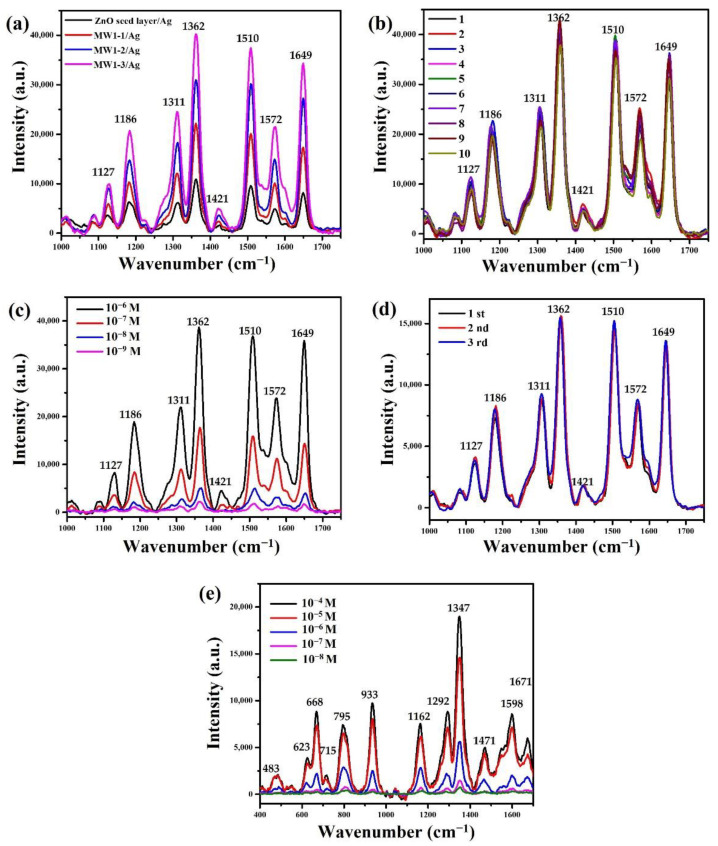
(**a**) SERS spectra of R6G (10^−6^ M) on ZnO/Ag nanoneedle arrays at the optimized temperature programming step. (**b**) The uniformity of SERS spectra of R6G (10^−6^ M) on ZnO/Ag nanoneedle arrays (MW1-3). (**c**) SERS spectra of R6G at different concentrations (10^−6^ M–10^−9^ M) were obtained from ZnO/Ag nanoneedle arrays (MW1-3). (**d**) The reusability of SERS spectra of R6G (10^−7^ M) on ZnO/Ag nanoneedle arrays (MW1-3). (**e**) SERS spectra of amoxicillin at different concentrations (10^−4^ M–10^−8^ M) were obtained from ZnO/Ag nanoneedle arrays (MW1-3).

The excellent geometric structure of the ZnO nanoneedle arrays can facilitate the uniform deposition of silver nanoparticles to form a three-dimensional surface-enhanced Raman scattering substrate. The low-and high-magnification TEM images (Figure S1a,b) of a ZnO/Ag nanoneedle were grown at the temperature programming step of MW1-3 and deposited 1 min Ag nanoparticles by an ion-sputtering method. As a result, the ZnO nanoneedle has been completely decorated with Ag nanoparticles. Two primary lattice fringes are observed on a ZnO/Ag nanoneedle in the HRTEM image (Figure S1c). First, the lattice fringes with a distance of 0.262 nm ascribed to the (002) plane of hexagonal ZnO crystal (JCPDS no. 89-0511). Second, the lattice fringes with a distance of 0.238 nm attributed to (111) plane of cubic Ag (JCPDS Card No.89-0722). Furthermore, the EDS mapping image (Figure S1d) of a ZnO/Ag nanoneedle is composed of Zn, O, and Ag and distributed with similar shapes. Figure 8a shows the SERS spectra of the ZnO nanoneedle arrays grown at the optimized temperature programming step with deposited 1 min Ag nanoparticles and immersed in the R6G solution (10^−6^ M) for 1h. The Raman characteristic peaks of the R6G molecule are around 1127, 1186, 1311, 1362, 1421, 1510, 1572, and 1649 cm^−1^, respectively, which can be attributed to C−H in-plane bending, C−O−C in-plane bending, and aromatic C−C stretch [46,47]. The intensity of all Raman peaks increases with the optimized temperature programming steps. This phenomenon is attributed to the construction of longer ZnO nanoneedle arrays that can deposit higher-density Ag nanoparticles with the highest SERS enhancement. The ZnO/Ag nanoneedle arrays (MW1-3) were used in the following experiments to further investigate the SERS performance due to its highest SERS enhancement.

In order to investigate the SERS uniformity of ZnO/Ag nanoneedle arrays, this study further measured the Raman spectra of R6G (10^−6^ M) at any ten different positions, as shown in Figure 8b. No significant change is observed from the position and intensity of the Raman peak at 1649 cm^−1^. This result demonstrates that ZnO/Ag nanoneedle arrays can be used for highly uniform SERS substrates. Figure 8c reveals the SERS spectra of R6G solution deposited on ZnO/Ag nanoneedle arrays at different concentrations (10^−6^ to 10^−9^ M). The intensities of all Raman peaks decreased with decreasing R6G solution concentration. The minimum detectable concentratin of R6G solution is 10^−9^ M. This result shows that ZnO/Ag nanoneedle arrays can be used for high-sensitivity SERS-based sensing. Figure 8d reveals the Raman spectra of the ZnO/Ag nanoneedle arrays with R6G (10^−7^ M) irradiated UV light before and after three cycles. The ZnO/Ag nanoneedle arrays reveal highly efficient reusability, and it still exhibits a similar Raman signal intensity after three times of repeated use.

To demonstrate that ZnO/Ag nanoneedle arrays can also be used for antibiotic detection. Herein, we selected the amoxicillin molecule as an antibiotic target. Amoxicillin is a bactericidal beta-lactam antibiotic drug molecule from the aminopenicillin family used to treat bacterial infections caused by susceptible bacteria [48,49]. The characteristic peaks of Raman spectra (Figure 8e) of the amoxicillin molecule are around 483, 623, 668, 715, 795, 933, 1162, 1292, 1347, 1471, 1598, and 1671 cm^−1^, respectively, which can be attributed to ring deformation in thiazole, bending in O−H, ring deformation in benzene, stretching in C−C, in-plane deformation in C−H, bending in NH_2_, C−H bending in benzene, in-plane deformation in benzene, twisting in NH_2_, asymmetric bending in CH_3_, stretching in benzene, and bending in N−H [50,51]. The intensities of all Raman peaks decreased with decreasing amoxicillin solution concentration. The minimum detectable concentration of amoxicillin solution is 10^−8^ M. This result shows that ZnO/Ag nanoneedle arrays can also be beneficial for detecting low-concentration antibiotics.

## 4. Conclusions

In summary, we have successfully fabricated ZnO nanoneedle arrays on the silicon substrate with the ZnO seed layer using microwave-assisted synthesis methods by appropriate temperature programming step under a short reaction time (25 min). The morphology of the ZnO nanoneedle arrays with a tapered shape and longer length exhibits excellent antireflection properties with a gradual decrease in the refractive index of Si substrate/ZnO/ambient air. Furthermore, ZnO nanoneedle arrays can deposit Ag nanoparticles with higher density and more hot spots in three-dimensional structures, providing high SERS enhancement, high uniformity, high reusability, and low detection limit for detecting R6G molecule. In addition, ZnO/Ag nanoneedle arrays can also use to detect low-concentration amoxicillin solution (10^−8^ M). Thus, the as-prepared ZnO nanoneedle arrays reveal potential applications for environmentally friendly and economical chemical or drug detection.

## Figures and Tables

**Table 1:** The different temperature programming steps (including heating time, reaction temperature, and holding time).

Sample	Heating Time (min)	Holding Time (min)	Heating Time (min)	Holding Time (min)	Heating Time (min)	Holding Time (min)
MW1-1	1 (80 °C)	4				
MW1-2	1 (80 °C)	4	1 (90 °C)	9		
MW1-3	1 (80 °C)	4	1 (90 °C)	9	1 (100 °C)	9
MW2	1 (80 °C)	25				
MW3	1 (80 °C)	4	1 (100 °C)	9	1 (120 °C)	9
MW4	1 (80 °C)	4	1 (90 °C)	14	1 (100 °C)	4
MW5	1 (80 °C)	4	1 (90 °C)	4	1 (100 °C)	14
MW6	1 (80 °C)	4	1 (85 °C)	4	1 (90 °C)	4
	1 (95 °C)	4	1 (100 °C)	4		
MW7	1 (80 °C)	4	1 (90 °C)	4	1 (100 °C)	4
	1 (110 °C)	4	1 (120 °C)	4		

## Data Availability

No new data were created or analyzed in this study. Data sharing is not applicable to this article.

## Supplementary Materials

The following supporting information can be downloaded at: https://www.mdpi.com/article/10.3390/nano12223989/s1, Figure S1: (a) low- and (b) high-magnification TEM, (c) HRTEM, and (d) EDS mapping images of a ZnO/Ag nanoneedle in Figure S1a. The EDS mapping images are Zn, O, and Ag, respectively. Figure S2: The ZnO nanoneedle arrays (MW1-3) lengths grown at different repeats.
