# Supplementary material for: Optimization Temperature Programming of Microwave-Assisted Synthesis ZnO Nanoneedle Arrays for Optical and Surface-Enhanced Raman Scattering Applications
Source: Nanomaterials (Basel). 2022 Nov 12;12(22):3989. doi: 10.3390/nano12223989 (PMC9696083; doi:10.3390/nano12223989)
Supplement: Supplementary file 1 [file nanomaterials-12-03989-s001.zip › nanomaterials-1968296-supplementary.pdf]

nanomaterials

Supplemental Information

Optimization Temperature Programming of Microwave-Assisted Synthesis ZnO Nanoneedle Arrays for Optical and Surface-Enhanced Raman Scattering Applications

Tung-Hao Chang [1,2,3], Yu-Cheng Chang [4,*], Chung-I Lee [5], Ying-Ru Lin [4,5], and Fu-Hsiang Ko [5,*]

[1] Department of Radiation Oncology, Changhua Christian Hospital, Changhua, 50006, Taiwan,
[2] Department of Radiological Technology, Yuanpei University, Hsinchu, 30015, Taiwan,
[3] Department of Medical Imaging and Radiological Sciences, Central Taiwan University of Science and Technology, Taichung, 40601, Taiwan,
[4] Department of Materials Science and Engineering, Feng Chia University, Taichung 407, Taiwan;
[5] Department of Materials Science and Engineering, National Yang Ming Chiao Tung University, Hsinchu 30010, Taiwan
* Correspondence: yuchchang@fcu.edu.tw, fhko@mail.nctu.edu.tw

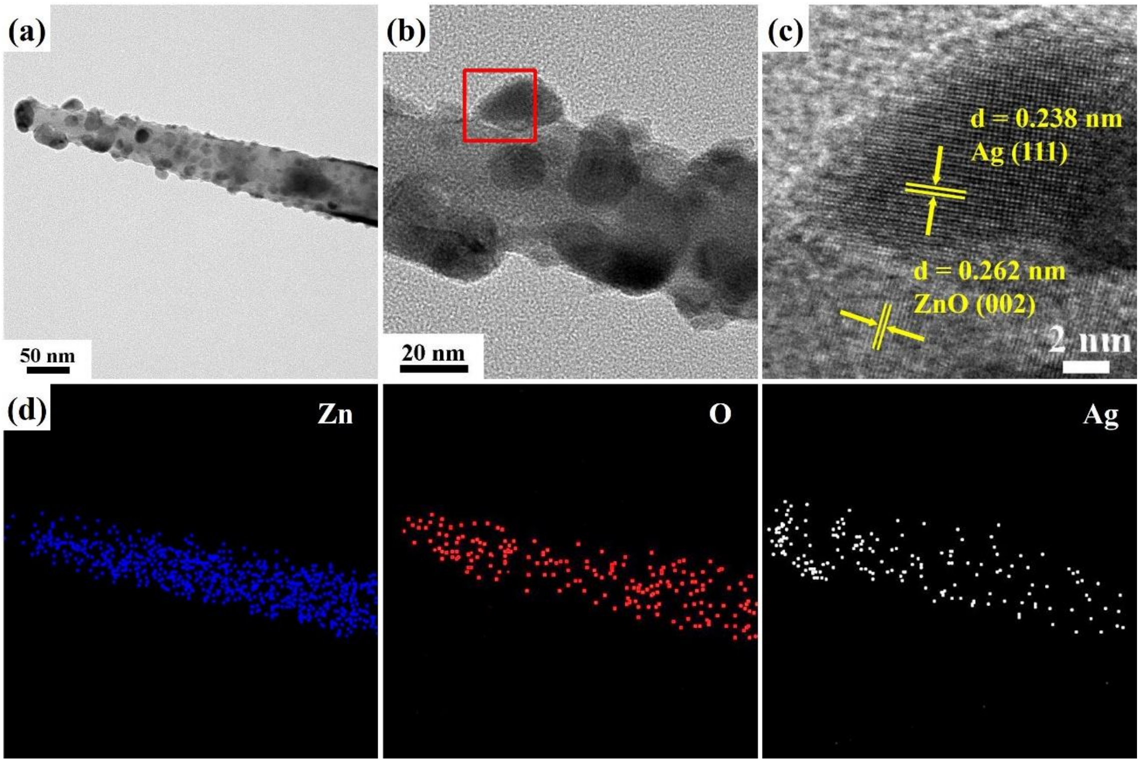

Figure S1 (a) low- and (b) high-magnification TEM, (c) HRTEM, and (d) EDS mapping images of a ZnO/Ag nanoneedle in Figure S1a. The EDS mapping images are Zn, O, and Ag, respectively.

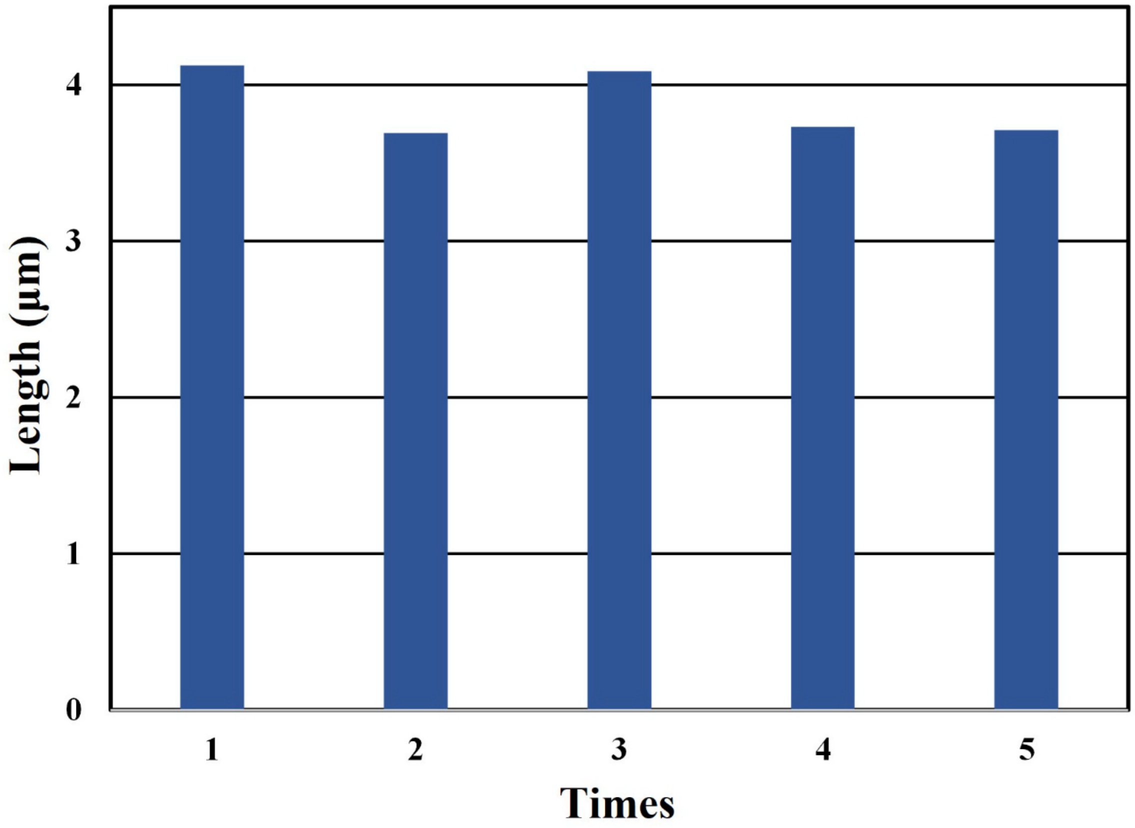

Figure S2 The ZnO nanoneedle arrays (MW1-3) lengths grown at different repeats.
